# Compression Therapy Challenges and Ulcer Recurrence in Chronic Venous Insufficiency: A Case Report and Review of Management Approaches

**DOI:** 10.7759/cureus.99902

**Published:** 2025-12-23

**Authors:** Rahul Ramakrishnan, Ahmik Shenoy, Ghaith Al-Eyd, Stephen Ely, Daniel Leon

**Affiliations:** 1 Medical Education, Nova Southeastern University Dr. Kiran C. Patel College of Allopathic Medicine, Fort Lauderdale, USA; 2 Medical Education, Arizona College of Osteopathic Medicine, Midwestern University, Glendale, USA; 3 Internal Medicine, Chen Senior Medical Center, Plantation, USA

**Keywords:** chronic venous insufficiency (cvi), compression therapy, elderly patient, patient education, ulcer staging, venous leg ulcer, venous reflex, ­wound healing

## Abstract

Chronic venous insufficiency (CVI) is a major cause of recurrent venous leg ulcers (VLUs) and presents treatment difficulties for elderly patients. This case report describes an 88-year-old female with CVI, cardiomyopathy, hypertension, dyslipidemia, and left-sided hemiplegia who presented with a 10-day history of left lower leg tenderness and purple-red discoloration. Examination revealed bilateral varicosities and a stage 4 VLU over the medial malleolus with purulent drainage and surrounding erythema. After 12 days of compression therapy, the ulcer improved to stage 3. However, two months of non-adherence to the treatment led to recurrence with a new stage 2 ulcer. The patient was subsequently initiated into a structured program with demonstrations, written instructions, scheduled reminders, and weekly follow-ups. After 22 days of consistent compression therapy, the ulcer improved to stage 1. This case emphasizes the importance of consistent compression therapy and multifaceted approaches in managing recurrent VLUs.

## Introduction

Chronic venous insufficiency (CVI) is characterized primarily by a sustained increase in venous pressure secondary to venous valve incompetency, venous obstruction, obesity, and immobility. Venous hypertension results in capillary distension, increased permeability, leakage of plasma proteins into the interstitium, resulting in activation of an inflammatory response. Subsequently, the inflammatory reaction damages the endothelium and capillary basement membranes, leading to fibrosis of the dermis and subcutaneous tissues, and tissue breakdown that ultimately results in ulcer formation. Common risk factors include advanced age, female sex, elevated body mass index, tobacco use, family history, sedentary lifestyle, malnutrition, and oral contraceptive use [1]. In particular, prolonged standing or prolonged sitting with dependent legs can increase venous hydrostatic pressure and are documented risk factors for venous reflux. Additionally, post-thrombotic syndrome, which can result after a deep vein thrombosis (DVT), is a strong predictor of long-term CVI. In its advanced stages, CVI can lead to venous leg ulcers (VLUs), which account for approximately 60-80% of all lower extremity ulcers [2]. These ulcers develop from increased venous pressure and subsequent damage to surrounding vessels.

Venous ulcer formation occurs through four stages [3]. Stage 1 presents with skin redness and inflammation of the subcutaneous skin. When this inflamed skin begins to leak fluid, the ulcer progresses to stage 2. If the skin presents with small, white patches of dead skin, the ulcer progresses to stage 3. Lastly, the ulcer is considered to be in stage 4 once the wound surface is formed. Duplex ultrasound is the diagnostic standard, with venous reflux characterized by more than 500 milliseconds of flow reversal in superficial veins and more than one second in deep veins. Duplex ultrasound should be completed by a qualified technologist in a certified vascular ultrasound laboratory with the patient in a standing position [4].

Published guidelines from multiple professional societies strongly recommend customized compression therapy (20-40 mmHg in the absence of arterial insufficiency) as the mainstay of CVI management and the primary treatment for VLUs. In particular, the Society of Vascular Surgery and the American Venous Forum recommend multicomponent (multilayer) compression bandaging over single-component compression for VLU management. These guidelines describe the multilayer bandage as consisting of layers of orthopedic wool padding, crepe bandage, elastic bandage, and an elastic outer bandage, which provides sustained compression. In contrast, single-component compression options, such as stockings or hose, are generally less effective than a multilayer compression system. Additional management measures also include aggressively monitored local wound care through effective dressing materials, debridement, and monitoring for and treatment of infections. Moreover, the management should also encompass control of contributing risk factors and consideration of more aggressive surgical/interventional therapies for refractory VLUs [5-7]. However, these guidelines acknowledge that adherence to treatment is a significant clinical challenge, highlighting the importance of patient education and close follow-up.

With an aging population and increased barriers to treatment adherence in elderly patients, primary care physicians may increasingly encounter VLUs as a complication of CVI. This case report aims to highlight the clinical challenges of managing recurrent VLUs in an elderly patient with CVI, emphasizing the importance of adherence, structured patient education, and consistent follow-up.

## Case presentation

**Figure 1:**
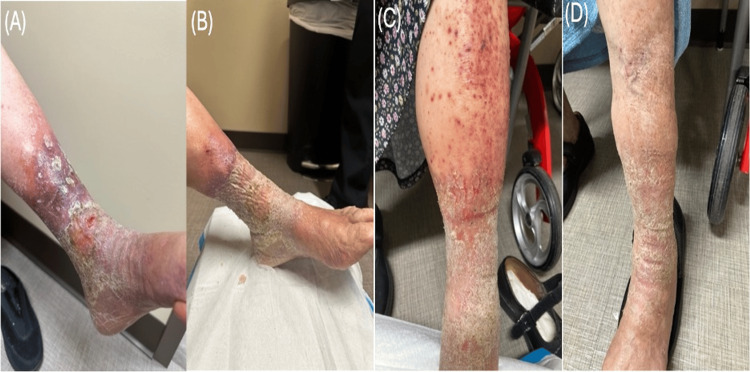
Physical examination findings. (A) Day 10 showing stage 4 venous leg ulcer (VLU). (B) Day 22 showing stage 3 VLU. (C) Day 101 showing a resolving VLU following strict compression therapy. (D) Day 104 showing stage 1 VLU following strict compression therapy.

An 88-year-old female with a long-standing history of CVI presented to the office with a 10-day history of tenderness and purple-red discoloration of the left lower leg. She reported a history of recurrent venous leg ulceration involving the left medial malleolus over several years, with multiple episodes per year. Prior ulceration was intermittently managed with local wound care and compression therapy. However, prior detailed records were unavailable, and the patient had inconsistent outpatient follow-up and adherence. The patient was unaware of the duration of her ulcer history. Her chronic symptoms include intermittent episodes of itching, swelling, and pain. Her medical history was also significant for cardiomyopathy, hypertension, dyslipidemia, and left-sided hemiplegia secondary to a prior cerebral infarction. She has had no previous DVT, but her baseline hemiplegia resulted in limited mobility. On examination, the patient appeared in moderate distress. Bilateral varicosities were noted along the shins and calves. On the left lower extremity, there was a stage 4 ulcer located over the medial malleolus measuring approximately 1 cm x 1 cm with a depth of 2 mm. The wound exhibited purulent discharge, debris, and mild granulation tissue. The surrounding skin showed erythema, purple-red discoloration, peeling extending to the mid-calf, induration, and tenderness upon calf palpation (Figure 1A). The affected limb was not warmer to the touch compared with the contralateral side, and the patient exhibited no signs of systemic infection. Prior to initiating compression therapy, arterial perfusion was evaluated clinically. Dorsalis pedis and posterior tibial pulses were palpable bilaterally, capillary refill was appropriate, and there were no clinical signs of peripheral arterial disease. Based on our physical examination findings, a formal ankle-brachial index (ABI) measurement was not obtained at the initial visit. As the patient’s clinical presentation was consistent with an ulcer secondary to advanced CVI and there were no concerning features for a DVT, vascular imaging was not performed at this initial evaluation.

The initial management plan included multicomponent compression therapy, leg elevation, and patient education regarding mobility and therapy adherence. At a follow-up visit on day 22 (12 days later), the ulcer had improved to stage 3, with a decreased size of approximately 0.8 x 0.8 cm, reduced drainage, and increased granulation tissue formation (Figure 1B). The patient was instructed to continue compression therapy indefinitely and return for follow-up every two weeks.

However, after approximately 60 days of non-adherence to multicomponent compression therapy, the patient re-presented on day 82 with increased tenderness, skin flaking, purple-red discoloration, and a new stage 2 VLU on the left lower leg measuring approximately 0.5 x 0.7 cm. In contrast to the initial management plan, the patient was subsequently provided with structured support, including scheduled reminders, visual aids, written instructions, and an additional demonstration on the correct application of compression stockings. Weekly follow-up appointments were scheduled, and further counseling was provided to emphasize the importance of continued long-term compression.

After 22 days of strict multicomponent compression therapy (day 104), the ulcer improved significantly, healing to a stage 1 lesion (Figures 1C, 1D). Regular follow-up was maintained to ensure proper technique of compression and to provide ongoing patient education.

## Discussion

Compression therapy with medical compression stockings remains the gold standard for the treatment and prevention of VLUs, with approximately 96% of patients achieving ulcer healing in one year [8]. The mechanism involves decreasing the diameter of superficial veins by applying an external graduated pressure to counteract any tissue expansion during calf muscle contraction [9]. Through this action, venous reflux and venous hypertension are reduced, which eventually increases venous return, decreases swelling, and allows ulcer healing. Multiple studies, reviews, and societal guidelines document the efficacy of compression therapies in the prevention and treatment of VLUs, particularly multilayer compression bandaging for stage 2 to stage 4 ulcers followed by medical compression stockings [5-10]. However, non-adherence to treatment has always been a major barrier to successful healing, especially among elderly patients. Our team’s management focused on a multicomponent compression bandaging.

In our case, objective clinical measurements such as wound dimensions and ulcer staging provided an effective way to evaluate disease severity and responses to treatment. These aspects showed improvement during periods of consistent compression and worsening during lack of adherence. Although duplex ultrasound remains a standard diagnostic test in assessing venous reflux, it was not performed during the initial evaluation. Instead, our team’s diagnosis relied on the chronic clinical features consistent with CVI, such as bilateral varicosities and lower extremity edema. This case reflects a real-world scenario in older adults with mobility limitations and emphasizes the importance of performing a thorough physical exam and planning a management regimen based initially on clinical features rather than immediate imaging.

Multiple interventional/surgical procedures to treat VLUs may be considered for specific indications to correct venous reflux, including ablation of incompetent superficial veins, ablation of the pathologic perforating veins, endophlebectomy, and valve transposition or transplantation.

Emerging research has explored novel approaches for managing CVI and promoting ulcer healing. One area of innovation involves the Surgical Anti-reflux Venous Valve Endoprosthesis (SAVVE) trial, which evaluated the VenoValve, a surgical bioprosthetic valve containing a porcine aortic valve. In Summer 2025, early trial results demonstrated that 84% of ulcers either healed or showed improvement, and that 91.6% of ulcers present for less than 12 months achieved complete healing [11]. However, in August 2025, the U.S. Food and Drug Administration (FDA) released a “Non-approvable” letter in response to the company’s Premarket Approval application [12]. Although the device shows promising early data, it is not approved for general patient use at this time.

Pharmacologic therapy also represents a promising area of research in CVI management. Hydroxyethylrutoside acts by limiting capillary permeability and edema, while pycnogenol appears to reduce passive venous dilation and vein stretching. A 2025 systematic review and meta-analysis suggested that both medications may improve CVI pain reduction and rating flux improvement. However, these findings were limited by sample size and study heterogeneity [13,14]. Following these studies, 2023 guidelines from the Society for Vascular Surgery, the American Venous Forum, and the American Vein and Lymphatic Society reported that the use of sulodexide, hydroxyethylrutosides, horse chestnut, calcium dobesilate, and red vine leaf extract may alleviate CVI-related pain, cramps, and heaviness [9]. In particular, the guidelines cited a systematic review in 2021 that found sulodexide to be as effective as pentoxifylline in promoting ulcer healing [15].

Beyond pharmacologic and procedural interventions, adherence to compression therapy remains the foundation of successful management. When evaluating LVUs, it is essential to consider other differentials such as arterial ulcers, diabetic ulcers, and pyoderma gangrenosum. Diagnosis of CVI and VLUs typically includes a detailed physical examination and duplex ultrasound to assess venous reflux. Recurrence remains a major concern, with studies showing that up to 80% of patients develop new ulcers within three months of healing [2]. Unfortunately, adherence to compression therapy is a constant challenge, with fewer than one-third of patients using compression as prescribed and approximately one-quarter utilizing compression only intermittently [16].

A limitation is that an official ABI measurement was not obtained prior to the initiation of compression therapy. Assessment of arterial perfusion with this measurement is generally recommended before compression to exclude significant peripheral arterial disease. In this patient, arterial perfusion was assessed clinically during the physical examination, and there was a positive response to compression therapy. However, the lack of the ABI measurement represents a limitation and highlights the need for using vascular assessment guidelines in clinical practice when managing patients with VLUs.

Maintaining adherence is difficult for elderly patients with limited mobility. A multifaceted approach that involves structured patient education, frequent follow-up, and multicomponent compression regimens can improve outcomes. Important strategies include demonstrating how to apply compression stockings, involving caregivers and family members, addressing patient concerns, and creating realistic patient-centered goals. In this case, the medical team increased compression therapy adherence through visual aids, demonstrations, scheduled reminders, and regular follow-up. These interventions improved patient understanding and consistency, allowing for successful ulcer healing and prevention of recurrence.

## Conclusions

This case emphasizes the importance of consistent compression therapy in the management and prevention of VLUs secondary to CVI. Non-adherence is a common cause of ulcer recurrence, particularly in elderly patients. Regular follow-up, continued patient education, and visually aided instructions are critical to ensuring adherence and preventing ulcer recurrence. Physicians should emphasize ongoing patient engagement, involving caregivers and family when appropriate, and consider various adherence interventions to improve outcomes and quality of life in patients with CVI.
